# Promoter methylation-associated loss of *ID4 *expression is a marker of tumour recurrence in human breast cancer

**DOI:** 10.1186/1471-2407-8-154

**Published:** 2008-05-30

**Authors:** Erik Noetzel, Jürgen Veeck, Dieter Niederacher, Oliver Galm, Felicitas Horn, Arndt Hartmann, Ruth Knüchel, Edgar Dahl

**Affiliations:** 1 Molecular Oncology Group, Institute of Pathology, University Hospital of the RWTH Aachen, Aachen, Germany; 2 Department of Gynaecology and Obstetrics, Heinrich-Heine University, Düsseldorf, Germany; 3 Medical Clinic IV, University Hospital of the RWTH Aachen, Aachen, Germany; 4 Department of Gynaecology and Obstetrics, University of Regensburg, Regensburg, Germany; 5 Institute of Pathology, University of Erlangen, Erlangen, Germany

## Abstract

**Background:**

Inhibitor of DNA binding/Inhibitor of differentiation 4 (*ID4*) is a critical factor for cell proliferation and differentiation in normal vertebrate development. *ID4* has regulative functions for differentiation and growth of the developing brain. The role of *ID1*, *ID2* and *ID3* are expected to be oncogenic due to their overexpression in pancreatic cancer and colorectal adenocarcinomas, respectively. Aside from these findings, loss of *ID3* expression was demonstrated in ovarian cancer. The aim of the present study was to reveal the factual role of *ID4* in carcinogenesis in more detail, since its role for the pathogenesis of human breast cancer has been discussed controversially, assigning both oncogenic and tumour suppressive functions.

**Methods:**

*ID4* promoter methylation, *ID4* mRNA expression and *ID4* protein expression were analysed in primary human breast cancer specimens using methylation-specific PCR (MSP) (n=170), semiquantitative realtime RT-PCR (n=46) and immunhistochemistry (n=3), respectively. In order to demonstrate a functional association of *ID4* promoter methylation with its gene silencing, we performed DNA demethylation analysis with four human breast cell lines using MSP and semiquantitative realtime RT-PCR. In addition, we performed correlations of *ID4* promoter methylation with *ID4* mRNA and *ID4* protein expression in matched samples of breast tumour and corresponding normal tissue. We carried out statistical analyses in order to find correlations between *ID4* promoter methylation and clinicopathological parameters.

**Results:**

Frequent *ID4* promoter methylation was observed in primary breast cancer samples (69%, 117/170). We found a tight correlation (P<0.0001) between *ID4* promoter methylation and loss of *ID4* expression in primary breast cancer 3 specimens. Demethylating treatment with breast cancer cell lines was associated with clear ID4 mRNA re-expression. Tumours with *ID4* promoter methylation showed distinct loss of *ID4* expression on both transcription and protein level. Interestingly, *ID4* promoter methylation was a factor for unfavourable recurrence-free survival (P=0.036) and increased risk for lymph node metastasis (P=0.030).

**Conclusion:**

ID4 is indeed a novel tumour suppressor gene in normal human breast tissue and is epigenetically silenced during cancer development, indicating increased risk for tumour relapse. Thus, *ID4* methylation status could serve as a prognostic biomarker in human breast cancer.

## Background

ID4 is the most recently discovered member of the Inhibitor of DNA binding/Inhibitor of differentiation family of transcription factors. ID proteins contain a helix-loop-helix (HLH) domain enabling interaction with other basic helix-loop-helix (bHLH)-proteins. Via hetero-dimerisation with those transcription factors, ID proteins act as dominant negative inhibitors of gene transcription [1]. In addition, ID proteins can also bind to some important non-bHLH transcription factors such as the *retinoblastoma gene *product (RB1) or the paired box (PAX)-proteins, thereby regulating important pathways in cell proliferation and differentiation [2,3]. Furthermore ID4 was found to be an important factor for the development of the nervous system. In this tissue, the *ID4 *gene is highly expressed in migrating postmitotic neurons, in Purkinje cells, as well as in the adult cerebellum. Since ID proteins regulate fundamental cellular processes, a link of ID dysregulation with human carcinogenesis has been recently postulated. *ID1*, *ID2 *and *ID3 *are overexpressed in several human tumour entities, e.g. pancreatic cancer [4] and colorectal adenocarcinomas [5]. In addition, *ID3 *showed decreased expression levels in several tumour types such as ovarian adenocarcinomas [6,7]. In contrast to the putative oncogenic properties of ID1 and ID2, ID4 expression was found to be decreased in a variety of human cancers [8]. Recently, it has become very evident that aberrant epigenetic modifications such as promoter methylation play a decisive role in the dysregulation of gene expression in cancer [9]. Hypermethylation of CpG-rich regions (CpG islands) in promoter sequences is an important mechanism for the silencing of tumour suppressor genes such as *p16*^*INKa*^, *p15*^*INK*4*b*^, *p14*^*ARF*^, *death-associated protein kinase (DAPK) *and *O-6-methylguanine-DNA methyltransferase (MGMT) *[10]. In breast cancer, a variety of critical genes were shown to be inactivated by methylation e.g. *BRCA1*, *14-3-3σ*, *TIM3 ESR1*, *PGR *and *E-cadherin *[11]. The *ID4 *promoter region contains also CpG islands which were found to be hypermethylated in gastric adenocarcinomas in association with gene silencing [12]. Several studies reported a potential correlation between *ID4 *promoter methylation and tumour initiation/progression, e.g. in colorectal carcinoma [13], human leukaemia [14] and prostate cancer [2]. In human breast tissue *ID4 *mRNA was found to be constitutively expressed in normal mammary epithelial cells, but suppressed in oestrogen receptor (ER)-positive breast carcinomas and pre-neoplastic lesions [15]. A human ribozyme library-based inverse genomics approach revealed that ID4 may act as a negative regulator of the common tumour suppressor gene *BRCA1 *[16,17]. Moreover, *ID4 *expression levels were found to be decreased in BRCA1/ER-positive breast cancer specimens, suggesting that ID4 participates in molecular events regulating ER and BRCA1 expression [18]. Aside from these expression data, a role of ID4 as a putative tumour suppressor in human breast cancer development has been discussed controversially and is uncertain yet. In contrast to the common ID4 downregulation in several human tumour entities, one study detected increased ID4 expression in rat mammary gland cells in conjunction with increased weight, proliferation and invasiveness of these tumours [19]. However, another study suggested that *ID4 *may act as tumour suppressor gene in a fraction of primary breast cancers, since aberrant hypermethylation of the *ID4 *gene promoter in T1 tumours was associated with an increased risk for lymph node metastasis [20]. In the present study, we readdressed the role of *ID4 *promoter methylation in human breast cancer development. To that end we analysed a large cohort (n = 170) of cryoconserved samples of breast cancer specimens, including all tumour sizes and histological grades. Using *in vitro *DNA demethylation treatment [21] of human breast cancer cell lines we wanted to determine whether *ID4 *promoter hypermethylation may affect *ID4 *mRNA transcription. Our next aim was to demonstrate for the first time a correlation between *ID4 *promoter methylation and loss of *ID4 *mRNA and protein expression in primary human breast cancer specimens. Finally, we aimed to analyse statistical correlations between clinicopathological patient characteristics and *ID4 *methylation and expression data.

## Methods

### Patient samples

Breast tissue samples (n = 170) used for methylation and mRNA expression analyses were obtained from patients treated by primary surgery for breast cancer at the Departments of Gynaecology at the University Hospitals of Aachen, Jena, Regensburg and Düsseldorf, Germany, with institutional review board approval. All patients gave informed consent to the study for retention and analysis of their tissue for research purposes. Part of the tumour material and macroscopically normal breast was snap-frozen in liquid nitrogen after surgery. Hematoxylin and eosin (H&E)-stained sections were prepared for assessment of the percentage of tumour cells, only samples with >70% tumour cells were selected. The normal breast tissue used for standardisation contained approximately 40% of epithelial cells. For patient characteristics see Table 1.

**Table 1:** Clinicopathological parameters of 170 breast cancer specimens analysed in this study.

**Variable**	**Categorisation**	***n*^***a ***^analysable**	**%**
***Clinicopathologic parameters:***			
Age at diagnosis:	median 57 years (range 28–87)		
	<50 years	53	31.2
	≥ 50 years	117	68.8
Tumour size^c^			
	pT1	62	36.5
	pT2-4	91	53.5
	pT x^b^	17	10
Lymph node status^c^			
	pN0	74	43.5
	pN1-3	62	36.5
	pN x	34	20
Histological grade			
	G1	14	8.2
	G2	79	46.5
	G3	61	35.9
	G x	16	9.4
Histological type			
	invasive ductal	128	75.3
	Invasive lobular	18	10.6
	other	24	14.1
Oestrogen receptor status			
	negative (IRS^d ^0–2)	52	30.6
	positive (IRS 3–12)	101	59.4
	IRS x	17	10
Progesterone receptor status			
	negative (IRS 0–2)	57	33.5
	positive (IRS 3–12)	96	56.5
	IRS x	17	10

^a^Only female patients with primary, unilateral, invasive breast cancer were included. ^b^x status unknown. ^c^According to TNM classification by Sobin and Wittekind [37]. ^d ^IRS= Immuno-reactive score according to Remmele and Stegner [38].

### Cell lines

The human breast cell lines BT20, MDA-MB231, MCF7 and T47D used for this study were obtained from the American Type Culture Collection (ATCC, Rockville, MD, USA) and cultured under recommended conditions.

### DNA/RNA isolation of breast cancer cells

Frozen tissue samples were dissolved in lysis buffer for subsequent DNA isolation using the blood and cell culture DNA kit (Qiagen, Hilden, Germany) or for RNA isolation by using TRIzol^® ^(Gibco-BRL, Glasgow, UK) according to the protocol supplied by the manufacturer.

### Reverse Transcription PCR

Of the total RNA, 1 μg was reverse transcribed using the Reverse Transcription System (Promega, Madison, WI, USA). To improve transcription rate we mixed oligo-dT and pdN_(6)_-Primers 1:2. For PCR, 1 μl cDNA was amplified using *ID4*, (For 5'-CGC TCA CTG CGC TCA ACA C-3' Rev 5'-CTA ACT TCT GCT CTT CCC CC-3', product size 148 bp) and *Glyceraldehyde-3-Phosphate Dehydrogenase *(*GAPDH*) (For 5'-GAA GGT GAA GGT CGG AGT CA-3' Rev 5'-TGG ACT CCA CGA CGT ACT CA-3', product size 289 bp) primers. Reactions were initiated as "Hot Start" PCR at 95°C for 5 min and held at 80°C before addition of 1 unit of *Taq *DNA polymerase (Roche, Mannheim, Germany). Cycle conditions applied for both genes were: 94°C for 5 min, 38 cycles of 95°C for 1 min, 58°C for 1 min, 72°C for 1 min and a final extension at 72°C for 10 min. PCR analyses were carried out in a PTC-200 cycler (Bio-Rad, formerly MJ Research, Hercules, CA, USA). The amplification products were analysed on a 2% agarose gel containing ethidium bromide under UV-light.

### Semi-quantitative realtime PCR

Semi-quantitative PCR was performed using the LightCycler system together with the LightCycler DNA Master SYBR Green I Kit (Roche, Mannheim, Germany) as previously described [22]. Reaction volumes of 20 μl consisted of the following components: 3 mM MgCl_2_, 10 μM forward primer, 10 μM reverse primer, 2 μl LightCycler DNA Master SYBR Green I and 1 μl of cDNA as PCR template. For primer sequences of *GAPDH *and *ID4 *amplification, see Reverse Transcription PCR section. To assure maximum specificity of *ID4 *mRNA detection a touchdown PCR program was designed (see additional file 1). Gene expression was quantified by the comparative CT method, normalising CT-values to the housekeeping gene *GAPDH *and calculating relative expression values [23]. Post amplification melting curve analyses were performed to assure product specificity. Relative *ID4 *expression levels were standardised in comparison to the expression level of pooled normal breast tissue samples. To ensure experiment accuracy, all reactions were performed in triplicates.

### Bisulphite-modification and methylation-specific PCR

Bisulphite-modification and methylation-specific PCR (MSP) were performed as previously described [22,24,25]. Of the genomic DNA, 1 μg was bisulphite-treated using the EZ DNA Methylation Kit™ (Zymo Research, Orange, CA, USA) according to the manufacturer's specifications. For MSP, 1 μl of modified DNA was amplified using MSP primers that specifically recognise the unmethylated (U For 5'-GGT AGT TG GAT TTT TTG TTT TTT AGT ATT-3' Rev 5'-AAC TAT ATT TAT AAA ACC ATA CAC CCC A-3, product size 157 bp) or methylated (M For 5'-TAG TCG GAT TTT TCG TTT TTT AGT ATC-3' Rev 5'-CTA TAT TTA TAA AAC CGT ACG CCC CG-3', product size 161 bp) *ID4 *promoter sequence after bisulphite conversion. DNA derived from human carcinoma cell line MDA-MB231 was bisulphite-treated to serve as a control for the unmethylated *ID4 *promoter sequence. DNA derived from human mammary carcinoma cell line BT20 was used as a positive control for methylated *ID4 *sequences as described elsewhere [20]. Amplification products were visualised by UV-illumination on 3% low range ultra agarose gel (Bio-Rad Laboratories, Hercules, CA, USA) containing ethidium bromide.

### 5-aza-2'-deoxycytidine (DAC) and trichostatin A (TSA) treatment

Cells were plated at a density of 3 × 10^4 ^cells/cm^2 ^in a 6-well plate on day 0. The demethylating agent DAC [26] (Sigma-Aldrich, Deisenheim, Germany) was added to a final concentration of 1 μM in fresh medium on days 1, 2 and 3. Additionally, 300 nM TSA (Sigma-Aldrich) was added on day 3. Cells were harvested on day 4 for RNA and DNA extraction. Control cells were incubated without the addition of DAC or TSA and fresh medium was supplied on days 1, 2 and 3.

### Immunhistochemistry (IHC)

Sections of three micrometers were dried for 30 min at 72°C, deparaffinised in xylene, rehydrated in a decreasing ethanol series and subsequently boiled for 35 min in Tris-EDTA buffer (pH 9.0) for antigen retrieval. Polyclonal ID4 rabbit anti-human antibody (Sc291, Santa Cruz, CA, USA) was utilised in 1:150 dilution and sections were incubated for 90 min. IHC was performed by using the ChemMate Envision Kit (DAKO, Hamburg, Germany). Sections were counterstained with Mayer's hematoxylin and embedded in Entellan^® ^mounting medium (EMS Diatome, Fort Washington, PA, USA). Sections of normal and tumorous colon tissues were used for positive controls as described elsewhere [13] (for details, see additional file 2). The application of primary antibody to tissue sections was omitted in negative controls.

### Statistical analyses of clinicopathological patient data

Statistical analyses were carried out by using SPSS version 14.0 (SPSS, Chicago, IL). Differences were considered statistically significant when P-values were located below 0.05. The two-sided, non-parametric Dunn's-Multiple-Comparison-Test was used in order to compare the *delta *CT values of the realtime RT-PCR results of the breast cancer group with the normal breast group as well as the different methylation groups. Two-sided Log-rank tests were performed in order to correlate RFS/OS with *ID4 *methylation and other clinicopathological parameters. A multivariate Cox-regression analysis was performed in order to test the independent prognostic relevance of *ID4 *methylation. The limit for reverse selection procedures was P = 0.2. The proportionality assumption for all variables was assessed with log-negative-log survival distribution functions.

## Results

### ID4 methylation, expression and re-expression analysis of mammary cell lines

**Figure 1:**
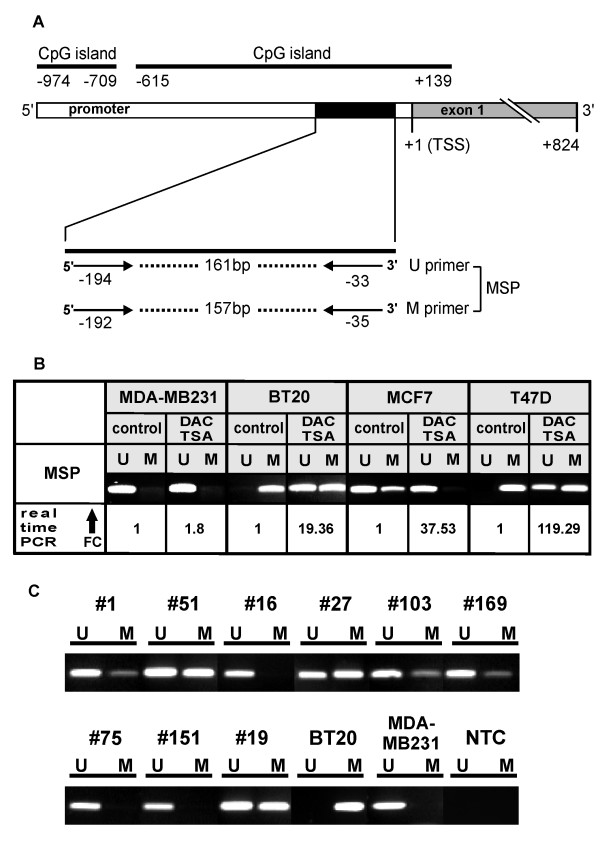
**(A) Illustration of the *ID4 *gene promoter region and of MSP primer topology**. Two CpG islands are located in the *ID4 *promoter region upstream of the transcription start site (TSS). Methylation-specific PCR (MSP) primers used for this study were positioned within the central CpG island (-615 until +139) near the TSS, detecting an amplicon indicated by the black box. The PCR product size of the unmethylated *ID4 *promoter sequence is 161 bp and similar in size to the product achieved with primers indicating methylated *ID4 *promoters (157 bp). The primers for the U reaction cover the bases -194 until -166 and -60 until -33. The primers for the M reaction cover the bases -192 until -166 and -60 until -35. All positions are relative to the TSS. **(B) *In vitro *demethylation analysis of the *ID4 *promoter in four human breast cancer cell lines**. Cells were incubated with 5-aza-2'-deoxycytidine (DAC) and trichostatin A (TSA) for 72 h and 24 h, respectively. For each cell line, the methylation status and the fold change (FC) of *ID4 *mRNA re-expression are shown. All methylated cell lines (BT20, MCF7 and T47D) restored *ID4 *expression after demethylating treatment. MDA-MB231 remains unmethylated in the *ID4 *promoter and exhibits only a marginal increase of *ID4 *mRNA expression after DAC/TSA treatment. **(C) *ID4 *promoter methylation analysis in primary human breast cancer (n = 170) using MSP technology**. MSP results from nine representative patients (#) are shown. DNA bands in lanes labelled with U indicate PCR products amplified with primers recognising the unmethylated promoter sequence. DNA bands in lanes labelled with M represent products amplified with primers specific for methylated alleles. Human breast cancer cell lines BT20 and MDA-MB321 were used as positive controls for methylated and unmethylated ID4 promoter sequences, as described previously [20]. Water was used as non template control (NTC).

First, we established a methylation-specific PCR (MSP) for the *ID4 *gene, using MSP primers which are complementary to the central CpG island of the *ID4 *promoter region (Figure 1A). The designed MSP primers amplify the *ID4 *promoter sequence starting approximately 30 bp upstream of the transcription start site (TSS). In order to demonstrate that *ID4 *promoter methylation may be associated with *ID4 *gene silencing, we performed demethylation analyses with four human breast cancer cell lines (MDA-MB231, BT20, MCF7 and T47D). For this purpose, these cell lines were treated with the demethylating agent DAC and the histone deacetylase inhibitor TSA. *ID4 *expression was measured 72 h later by performing realtime PCR (Figure 1B). We found that in all methylated cell lines (BT20, MCF7, and T47D) *ID4 *mRNA expression was restored after the treatment. The increase of *ID4 *expression after promoter demethylation was 119-fold in T47D cells, 38-fold in MCF7 cells and 19-fold in BT20 breast cancer cells. The unmethylated cell line MBA-MD231 showed just a marginal alteration of its *ID4 *mRNA levels.

### ID4 promoter methylation in primary human breast cancer

Recently we have demonstrated that *ID4 *mRNA expression is downregulated in 78% (39/50) of human primary breast carcinomas [27]. Umetani et al. had shown before that promoter hypermethylation is implicated to be an effective mechanism of *ID4 *inactivation in human breast cancer, albeit this group only analysed small sized (T1) breast tumours [20]. In order to determine the exact methylation frequency of the *ID4 *promoter in a clinical relevant spectrum of human breast cancer we analysed genomic DNA from 170 primary breast cancer patients by MSP technology.

Representative results are shown in Figure 1C. In total *ID4 *promoter methylation was found in 68.9% (117/170) of breast cancer specimens. Accordingly, 31.1% of the breast cancer specimens (53/170) exhibited no *ID4 *promoter methylation. Normal breast tissues (n = 13) were analysed by MSP as well and did not exhibit any *ID4 *promoter methylation (data not shown), indicating that this is a tumour-specific process.

### Correlation analyses between ID4 promoter methylation and ID4 expression in human breast cancer

**Figure 2:**
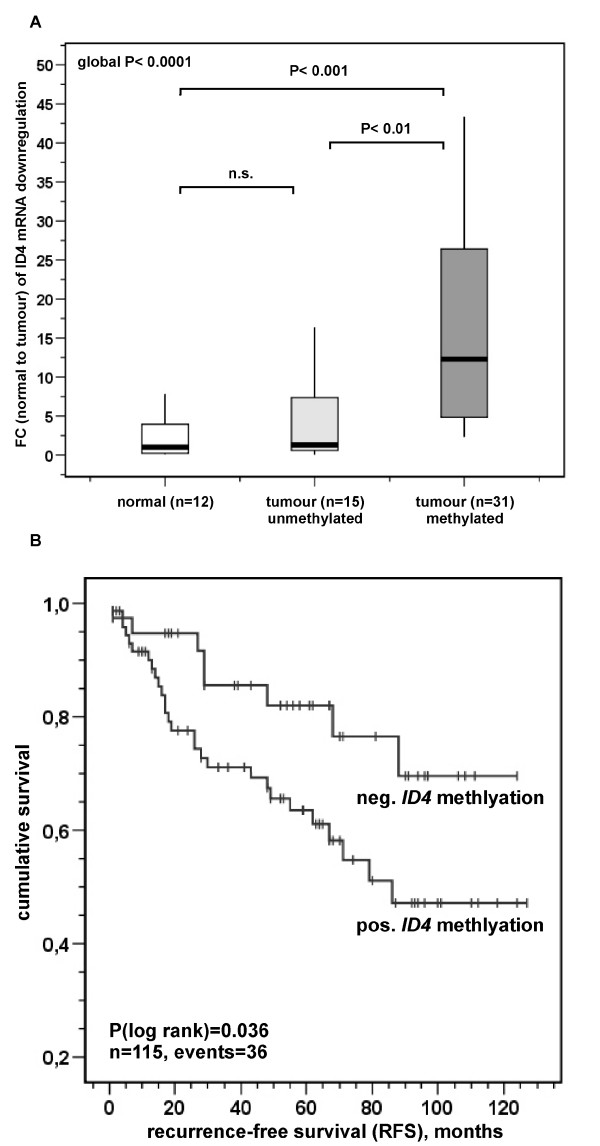
**(A) Correlation between *ID4 *expression and *ID4 *promoter methylation in human breast cancer**. Box plot analysis illustrating the loss of *ID4 *expression in relation to *ID4 *promoter methylation in primary human breast cancer. The Y axis indicates the factor of *ID4 *mRNA downregulation in breast cancer specimens (n = 46) relative to a normal breast standard (a pool of 12 normal breast tissue samples) as the fold change (FC) N/T. Unmethylated tumours exhibited ID4 expression (median FC = 1.3) very similar to normal breast cells. In contrast, methylated breast cancer specimens displayed an increased loss of *ID4 *expression (median FC = 12.3). Horizontal lines: group medians; boxes: 25–75% quartiles; vertical lines: range, peak and minimum. **(B) Kaplan-Meier analysis of patients' recurrence-free survival (RFS) in relation to *ID4 *promoter methylation**. Distribution of time (months) and tumour-related death among 115 breast cancer patients with positive (lower graph) or negative (upper graph) *ID4 *promoter methylation state is shown. Patients harbouring an *ID4*-methylated tumour have an estimated mean RFS time of 80 months (95% confidence interval: 67–93 months) compared with 101 months (95% confidence interval: 87–115 months) for patients without *ID4 *tumour methylation. See text for details.

**Figure 3:**
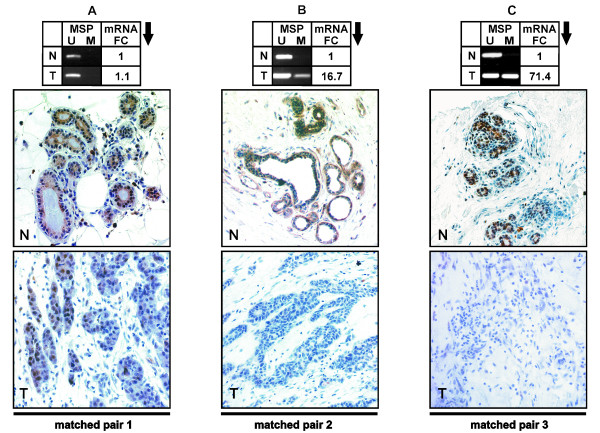
**Correlation of *ID4 *promoter methylation with ID4 expression on both mRNA and protein level**. Coherence of *ID4 *promoter methylation and ID4 expression in three representative samples of primary breast cancer and corresponding normal tissue (matched pairs a, b, c). Normal tissues are labelled with N. Their corresponding tumours are labelled with T. For each sample, *ID4 *promoter methylation, *ID4 *mRNA expression and ID4 immunohistochemical staining are shown (original magnification: 400×). The Fold change (FC) of *ID4 *mRNA downregulation is calculated by the expression ratio of normal (N) and tumourous tissue (T) for each matched pair (a, b, c). Matched pair a: Unmethylated tumour showed very similar *ID4 *mRNA and protein expression compared to the corresponding normal breast tissue. Matched pairs b and c: Methylated tumours exhibited explicit loss of *ID4 *mRNA and ID4 protein in comparison to the corresponding normal breast tissues (N). See text for details.

Next, we wanted to analyse whether *ID4 *promoter methylation consequently led to silencing of the *ID4 *promoter as measured by realtime PCR analysis of the gene transcript. For this purpose, a part of the same breast cancer cohort (n = 46) used previously for methylation analysis was re-assessed (Figure 2A). Compared to a normal breast tissue standard (12 pooled normal breast tissues) loss of *ID4 *mRNA expression in unmethylated breast cancer specimens was marginal (median fold change = 1.3). In contrast, methylated breast cancer specimens exhibited a highly significant (P < 0.001) loss of *ID4 *expression (median fold change = 12.3). Thus, these data clearly indicate that *ID4 *promoter methylation is associated with *ID4 *gene silencing. The comparison of *ID4 *expression in breast tumours versus normal breast tissues resulted in 82.6% downregulation in tumour samples by the fold change two (FC 2) approach. In order to confirm that promoter methylation also affects loss of ID4 protein, we performed a parallel analysis of *ID4 *promoter methylation, mRNA and protein expression in three matched samples with normal breast tissue and corresponding tumour tissue (Figure 3A, B, C). Breast cancer specimens (T) with unmethylated *ID4 *promoter exhibited only a marginal decline (FC = 1.1) in *ID4 *mRNA expression. In accordance with the mRNA data, the abundance of ID4 protein in the tumour was very similar to that found in the corresponding normal tissue (N). Breast cancer specimens exhibited strong *ID4 *mRNA downregulation (16-fold and 71-fold, respectively) in comparison to their corresponding normal tissues depending on clear *ID4 *promoter methylation. Note, that in these tumour tissues nearly complete loss of ID4 protein expression was evident (Figure 3B, C).

### Statistical analysis of clinicopathological patient data and patient survival

Finally, descriptive Fisher's exact tests were performed in order to correlate *ID4 *methylation with clinicopathological patient characteristics (Table 2). A hypermethylated *ID4 *promoter was significantly associated with positive lymph node status (P = 0.03) and loss of *ID4 m*RNA expression (P < 0.001). No associations were found with age at diagnosis, tumour size, histological grade/type and oestrogen/progesterone receptor status. A comparison between recurrence-free survival (RFS)/overall survival (OS) and *ID4 *methylation status is shown in Table 3. We found an increased risk for tumour recurrence in breast cancer patients with *ID4 *promoter methylation (P = 0.036) compared to patients with lack of *ID4 *methylation (Figure 2B). Estimation was accomplished by the method of Kaplan-Meier. *ID4 *promoter methylation is considerably associated with a low 10-years RFS rate (47%) while patients without *ID4 *promoter methylation have a 10-years RFS rate of 71%. Cox regression models including factors possibly influencing RFS in relation to *ID4 *promoter methylation, failed significance in confirming the prognostic value of *ID4 *promoter methylation as an independent marker, probably due to its close relation to positive lymph node status (data not shown).

**Table 2:** Clinicopathological parameters in relation to *ID4 *promoter methylation

**Variable**	***ID4 *Methylation**			
	
	**n**^**a**^	**Positive**	**Negative**	**P-value**^**d**^
*Total*	170	117	53	
***Clinicopathological parameters:***				
Age at diagnosis				
<50 years	51	34	17	0.821
≥ 50 years	111	76	35	
Tumour size^b^				
pT1	62	24	44	0.247
pT2-4	91	27	7	
Lymph node status^b^				
pN0	74	43	31	**0.030**
pN1-3	62	47	15	
Histological grade				
G1	14	10	4	0.944
G2	79	52	27	
G3	61	42	19	
Histological type				
invasive ductal	129	87	42	0.940
invasive lobular	18	12	6	
other	12	8	4	
Oestrogen receptor status				
negative (IRS^c ^0–2)	52	35	17	0.900
positive (IRS 3–12)	101	69	32	
Progesterone receptor status				
negative (IRS 0–2)	57	39	18	0.920
positive (IRS 3–12)	96	65	31	
*ID4 *mRNA expression x-fold				
FC<2	8	0	8	**<0.001**
FC≥2	38	31	7	

^a ^Only female patients with primary, unilateral, invasive breast cancer were included. Significant P- values are marked in bold face. ^b^According to TNM classification by Sobin and Wittekind [37]. ^c ^IRS = Immuno-reactive score according to Remmele and Stegner [38]. ^d^Fisher's exact test. Significant P- values are marked in bold face.

**Table 3:** Univariate analysis of clinicopathological parameters influencing recurrence-free survival (RFS) and overall survival (OS)

**Variable**	**RFS**			**OS**		
	
	**n**^**a**^	**events**	**P-value**^**d**^	**n**	**events**	**P-value**
***Clinicopathological factors:***						
Tumour size^b^						
pT1	44	14	0.142	45	7	0.396
pT2-4	77	35		79	16	
Lymph node status^b^						
pN0	55	11	**0.021**	54	6	**0.033**
pN1-3	59	24		59	15	
Histological grade						
G1	11	3	**0.018**	10	1	**0.019**
G2	60	15		58	7	
G3	60	30		54	17	
Histological type						
invasive ductal	107	33	**0.006**	106	23	0.877
invasive lobular	18	9		18	2	
other	9	7		9	2	
Oestrogen receptor status						
negative (IRS^c ^0–2)	46	15	0.701	45	12	**0.027**
positive (IRS 3–12)	82	32		82	14	
Progesterone receptor status						
negative (IRS^c ^0–2)	43	18	0.240	43	11	0.059
positive (IRS 3–12)	85	29		84	15	
*ID4 *promoter methylation						
negative	39	8	**0.036**	39	5	0.169
positive	76	28		75	16	

^a^Only female patients with primary, unilateral, invasive breast cancer were included. ^b^According to TNM classification by Sobin and Wittekind [37]. ^c ^IRS = Immuno-reactive score according to Remmele and Stegner [38]. ^d^Fisher's exact test. Significant P-values are marked in bold face.

## Discussion

Previous studies have shown that the HLH transcription factor ID4 is functionally associated with fundamental processes such as differentiation, proliferation, apoptosis and angiogenesis [3] via interaction with cell cycle factors like RB1-protein or the PAX-proteins [28]. For this reason it is not surprising that all ID family members have been reported to be dysregulated in several human tumour entities [29].

Epigenetic inactivation of the *ID4 *gene through promoter methylation has been shown for several human tumour types such as gastric carcinoma [12], colorectal carcinoma [13] and acute leukaemia [14]. In breast cancer the epigenetic regulation of *ID4 *expression was demonstrated in 67% (16/24) of node positive tumours, although only breast tumours of small size (T1 tumours) were analysed in this study [20]. Thus, it was the aim of the present work to analyse the role of *ID4 *promoter methylation in a clinical relevant cohort of human breast cancer and further to study this process in human cell lines. *ID4 *promoter methylation is indeed associated with *ID4 *gene silencing in human breast cancer cell lines as *in vitro *demethylation experiments with DAC in three methylated breast cancer cell lines restored abundant *ID4 *mRNA expression. These cell line results represent the prerequisite for a putative tumour suppressive role of *ID4 *promoter methylation in human breast cancer. Up to now, epigenetic silencing of *ID4 *has been demonstrated only for gastric adenocarcinoma [12] and colorectal carcinoma cell lines [13]. In addition, we could show that a high percentage of human primary breast cancers (69%) exhibit hypermethylation of the *ID4 *promoter. Furthermore, we could show that *ID4 *promoter methylation in human breast cancer is significantly associated with loss of *ID4 *mRNA expression, this tight correlation again being a prerequisite for a putative tumour suppressive function of *ID4 *promoter methylation in human breast cancer. Our results demonstrate a highly significant loss of *ID4 *mRNA in 83% of human breast cancers. This incidence of *ID4 *expression loss is very similar to the 78% of *ID4 *mRNA downregulation measured previously by a cancer profiling array [27]. However, our findings are not in accordance with the determined *ID4 *mRNA upregulation described for rat breast carcinoma cells [19]. Further studies will have to show, whether ID4 regulation in human and rat breast carcinogenesis might differ.

Statistical analysis furthermore revealed that *ID4 *promoter methylation represents an adverse prognostic factor. Breast cancer patients harbouring a methylated *ID4 *promoter were found to have a decreased mean RFS time in comparison to patients without *ID4 *methylation in the tumour, supporting the hypothesis that a functional *ID4 *gene indeed confers tumour suppressive functions to human breast tissue. Thus, ID4 may have the opposite function of ID1 and ID2, which are thought to have oncogenic properties in human breast cancer cells [30,31]. Additionally, Perk et al. reported an increased ID1 expression in human bladder and prostate cancer [32]. Supporting a metastasis suppressing function of ID4, we found a significant positive correlation between *ID4 *promoter methylation and lymph node metastasis in our large cohort (n = 170) of breast cancer patients. This correlation was also suggested for the cohort of T1 tumours in the study of Umetani et al. [20]. No further correlations between *ID4 *methylation and other clinicopathological parameters were found. To our knowledge, this is the first study presenting a distinct loss of ID4 protein expression and *ID4 *mRNA downregulation associated with *ID4 *promoter hypermethylation in human breast cancer. The loss of protein expression, which modulates the activity of its downstream targets, is an important milestone for the validation of ID4 as a novel TSG in human breast cancer. Up to now loss of the ID4 protein expression was observed in sporadic breast adenocarcinomas [33] and colorectal carcinomas [13].

However, in these studies correlations between *ID4 *methylation and *ID4 *transcription were not determined. In conclusion, our data show that *ID4 *is a potential tumour suppressor gene in breast cancer that becomes epigenetically inactivated during cancer development owing to aberrant promoter methylation. Our investigations form a basis for further functional analyses in order to light up the importance of *ID4 *for the progression and metastasis of human breast cancer. The inactivation of tumour suppressor genes through promoter methylation offers new opportunities to identify novel DNA biomarkers in human cancer diseases that may also represent targets for improved future therapies [34,35]. DNA methylation marker panels promise early detection, risk assessment, chemoprediction and monitoring for disease recurrence in combination with a minimally/non-invasive detection in the blood stream or from archived tissue specimens [36].

## Conclusion

In summary, our analyses regarding aberrant ID4 promoter methylation and differential ID4 expression on both mRNA and protein level lead to the following conclusions: ID4 is indeed a tumour suppressor gene in normal breast tissue, which undergoes epigenetical silencing during breast tumour development. The methylation status of ID4 predicts early tumour relapse and could serve as a prognostic biomarker in human breast cancer.

## Competing interests

The authors declare that they have no competing interests.

## Authors' contributions

EN participated in the design of the study, carried out the experimental data acquisition, performed data analyses/interpretation and drafted the manuscript. JV processed clinical samples for realtime PCR and MSP analyses, participated in the design of the study and critically revised the manuscript. DN provided patient material and clinicopathological data and critically revised the manuscript. FH provided patient material and clinicopathological data and critically revised the manuscript. AH provided patient material and clinicopathological data and critically revised the manuscript. OG participated in the design of MSP analyses and critically revised the manuscript. RK participated in the design and coordination of the study, supplied administrative support and critically revised the manuscript. ED designed and coordinated the study and critically revised the manuscript. All authors read and approved the final manuscript.

## Pre-publication history

The pre-publication history for this paper can be accessed here:



## Supplementary Material

Additional file 1 "Touchdown PCR for *ID4 *mRNA expression analysis" Click here for file

Additional file 2 **"Positive controls for the immunohistochemical staining with normal and tumourous colon tissues for a polyclonal ID4 antibody (sc-491)." **A) and C) are negative controls without ID4 antibody incubation for normal and tumourous colon tissues, respectively. B) Intensive cytoplasmatic staining of a normal colon tissue. D) Negative ID4 protein staining for an infiltrating colon carcinoma (G2, pT2, pN0, pMx). Click here for file
