# Supplementary material for: Promoter methylation-associated loss of ID4 expression is a marker of tumour recurrence in human breast cancer
Source: BMC Cancer. 2008 May 30;8:154. doi: 10.1186/1471-2407-8-154 (PMC2435120; doi:10.1186/1471-2407-8-154)
Supplement: Additional file 1 — "Touchdown PCR for ID4 mRNA expression analysis" [file 1471-2407-8-154-S1.pdf]

Additional file 1. Touchdown PCR for *ID4* mRNA expression analysis.

Step	***Temperature***	***Time***	***Quantity***
Initial DNA Denaturation	95°C	5 min.	1
Denaturation<br>Primer Annealing<br>Elongation (DNA-Synthesis)	95°C<br>71°C<br>72°C	20 sec.<br>5 sec.<br>4 sec.	5
Touchdown to target temperature of 65°C (stepwise)	1°C per Cycle		6
Denaturation<br>Primer Annealing<br>Elongation (DNA-Synthesis)	95°C<br>65°C<br>72°C	20 sec.<br>5 sec.<br>4 sec.	28
Final Elongation (DNA-Synthesis)	72°C	60 sec.	1
