# Supplementary material for: Promoter methylation-associated loss of ID4 expression is a marker of tumour recurrence in human breast cancer
Source: BMC Cancer. 2008 May 30;8:154. doi: 10.1186/1471-2407-8-154 (PMC2435120; doi:10.1186/1471-2407-8-154)
Supplement: Additional file 2 — "Positive controls for the immunohistochemical staining with normal and tumourous colon tissues for a polyclonal ID4 antibody (sc-491)." A) and C) are negative controls without ID4 antibody incubation for normal and tumourous colon tissues, respectively. B) Intensive cytoplasmatic staining of a normal colon tissue. D) Negative ID4 protein staining for an infiltrating colon carcinoma (G2, pT2, pN0, pMx). [file 1471-2407-8-154-S2.pdf]

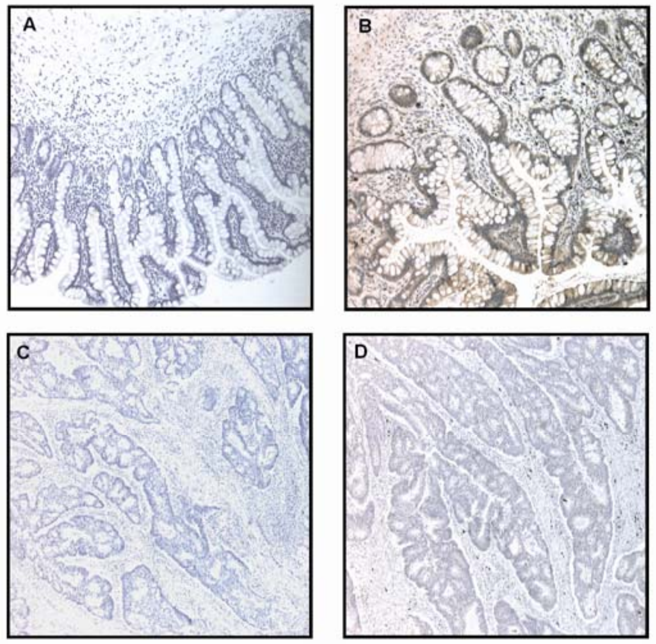

Additional file 2: Positive controls for the immunohistochemical staining with normal and tumourous colon tissues for a polyclonal ID4 antibody (sc-491). A) and C) are negative controls without ID4 antibody incubation for normal and tumourous colon tissues, respectively. B) Intensive cytoplasmatic staining of a normal colon tissue. D) Negative ID4 protein staining for an infiltrating colon carcinoma (G2, pT2, pN0, pMx)."
